# Lipoprotein apheresis results in plaque stabilization and prevention of cardiovascular events: comments on the prospective Pro(a)LiFe study

**DOI:** 10.1007/s11789-015-0068-y

**Published:** 2015-02-03

**Authors:** Reinhard Klingel, Andreas Heibges, Cordula Fassbender

**Affiliations:** 1 Apheresis Research Institute, Stadtwaldgürtel 77, 50935 Cologne, Germany; 2 1st Department Internal Medicine, University of Mainz, Langenbeckstr. 1, 55131 Mainz, Germany

**Keywords:** Lipoprotein apheresis, Lipoprotein(a), Cardiovascular disease, Plaque stabilization, Coronary artery disease, Prevention, Lipoprotein-Apherese, Lipoprotein(a), Kardiovaskuläre Erkrankung, Plaque Stabilisierung, Koronare Herzerkrankung, Prävention

## Abstract

Elevated lipoprotein(a) (Lp(a)) has emerged as an important independent cardiovascular risk factor, and causal association has been accepted with adverse outcome in atherosclerotic disease. Lipoprotein apheresis (LA) can lower low-density lipoprotein (LDL)-cholesterol and Lp(a) by 60–70 % and is the final escalating therapeutic option in patients with hyperlipoproteinemias (HLP) involving LDL or Lp(a) particles. Major therapeutic effect of LA is preventing cardiovascular events. Stabilizing plaque morphology might be an important underlying mechanism of action. In Germany, since 2008, a reimbursement guideline has been implemented to establish the indication for LA not only for familial or severe forms of hypercholesterolemia but also for Lp(a)-HLP associated with a progressive course of cardiovascular disease, that persists despite effective treatment of other concomitant cardiovascular risk factors, i.e. isolated Lp(a)-HLP. The Pro(a)LiFe-study confirmed with a prospective multicenter design that LA can effectively reduce Lp(a) plasma levels and prevent cardiovascular events.

## Abstract (de)

Ein erhöhter Spiegel von Lipoprotein(a) (Lp(a)) ist ein bedeutender unabhängiger und kausal zu wertender kardiovaskulärer Risikofaktor. Die Lipoprotein-Apherese (LA) kann LDL-Cholesterin und Lp(a) um 60–70 % absenken und ist die ultima ratio der Therapie-Eskalation bei Hypercholesterinämie und Lp(a)-Hyperlipoproteinämie (Lp(a)-HLP). Der wesentliche therapeutische Effekt der LA besteht in der Prävention kardiovaskulärer Ereignisse. Die Stabilisierung der Plaquemorphologie könnte hierbei ein wichtiger Wirkmechanismus sein. Die deutschen Erstattungsrichtlinien für die LA umfassen seit 2008 nicht nur die familäre und schwere Hypercholesterinämie sondern auch die Lp(a)-HLP mit progredienter Gefäßerkrankung, die persistiert trotz effektiver Behandlung aller bestehenden kardiovaskulären Risikofaktoren und dann als isolierte Lp(a)-HLP bezeichnet wird. Die Pro(a)LiFe-Studie bestätigte mit prospektivem Multicenter-Design, daß die LA zur effektiven Reduktion der Lp(a)-Spiegel und Prävention kardiovaskulärer Ereignisse führte.

## Background

It lasted nearly 50 years after first description in 1963 until elevated lipoprotein(a) (Lp(a)) has been accepted as an important independent and causal risk factor associated with adverse outcome in atherosclerotic disease [1]. Part of the long-term misconceiving of Lp(a) were problems with laboratory measurement. In 1993, analysis of the physicians’ health study concluded on no evidence of association between Lp(a) level and risk of future coronary artery disease [2]. Then it became clear that the enzyme-linked immunosorbent assay used in the study was susceptible to freezing artifacts [3]. Re-evaluation of the study revealed that Lp(a) predicted risk of coronary artery disease [4]. The physiological role of Lp(a) still remains to be fully elucidated. Pathogenic mechanisms linked to elevated Lp(a) levels can potentially increase the risk of cardiovascular disease (CVD) via prothrombotic or antifibrinolytic effects and via accelerated atherogenesis as a result of intimal deposition of Lp(a), or both [1].

Lipoprotein apheresis (LA) is the final escalating option to lower blood low-density lipoprotein cholesterol (LDL-C) levels in severely hypercholesterolemic patients including familial hypercholesterolemia, resistant to or intolerant of lipid lowering medication. Since 1991, regular reimbursement of LA has been implemented in guidelines of statutory health insurance funds in Germany. Ability of LA methods to lower Lp(a) as effective as LDL-C led to encouraging pilot experiences in a small number of patients with Lp(a)-hyperlipoproteinemias (HLP) and exceedingly progressive CVD in the early 1990s. In 2008, German Federal Joint Committee (GBA) decided to accept Lp(a)-HLP as separate indication for chronic LA. Lp(a)-HLP must have Lp(a) levels > 60 mg/dl and should be isolated in the sense that all other cardiovascular risk factors in particular LDL-C have to be under optimized treatment [5]. Lp(a) levels are generally not influenced by lifestyle. Nicotinic acid at high doses has shown Lp(a) lowering but was withdrawn in Europe in 2013. GBA stipulated with the new reimbursement guideline that additional prospective data are required to justify its maintenance. A longitudinal cohort study for the first time documented the therapeutic potential of LA to prevent cardiovascular events in this subgroup of CVD patients before the current reimbursement guideline was implemented [6]. These results strongly supported ethical concerns to withhold LA to such high-risk patients in a randomized controlled trial, which consequently could not achieve ethics committee approval. The best possible way to generate new prospective data in this situation was conduct of a prospective cohort study comparing the incidence of cardiovascular events in patients with Lp(a)-HLP and progressive CVD retrospectively before and prospectively after commencing chronic LA. Two-year results of the 5-year prospective Pro(a)LiFe-study have been published [7].

## Patients

The German reimbursement guideline permits LA for patients with Lp(a) > 60 mg/dl, LDL-C in normal range, and persisting progressive CVD in coronary, peripheral, or cerebral vascular beds. No further explanations were specified by GBA how to define normal range of LDL-C and progression of CVD or what other clinical conditions should be exhibited by candidates for LA. Hurdles for approval of chronic LA have been set high resulting in very select patients. According to current practice, the following conditions carry weight for assessing the individual risk profile and approval of LA by committees of regional associations of statutory health insurance physicians: progressive CVD as documented clinically and with imaging techniques, established maximally tolerated lipid-lowering drug treatment, recent cardiovascular events despite efficient drug treatment, out-of-the-ordinary frequency of cardiovascular events, early CVD in the patient, or positive family history of early CVD. It should be noted, that from the clinical point of view an acute event alone would not fulfill requirements of the guideline, on the other hand a recent acute event is no prerequisite for approval.

## Study design

Design of a prospective observational study was chosen for Pro(a)LiFe comparing the incidence of cardiovascular events in patients with Lp(a)-HLP and progressive CVD with a predefined uniform observation period retrospectively before and prospectively after commencing chronic LA. The past 2 years prior to commencing LA were selected as the major period of retrospective analysis, as they should have the best quality of retrospective data. Additionally, time of diagnosis and times of first and second cardiovascular events were documented. The first 5 years after commencing LA were selected for prospective analysis. Results of the first cohort study were used as guidance for sample size estimation [6, 7].

### Threshold of 60 mg/dl for Lp(a) in the German reimbursement guideline for LA

Lp(a) is a plasma lipoprotein consisting of a cholesterol-rich LDL particle with one molecule of apolipoprotein B100 and an additional apolipoprotein(a) (apo(a)) molecule. Apo(a) contains ten different types of plasminogen kringle 4-like repeats as well as regions homologous to the kringle 5 and protease-P of plasminogen. The kringle 4 type 2 domain is present in multiple repeated copies that differ in number (2 to > 40) between apo(a) isoforms [1]. Additionally, cholesterol, triglyceride, and phospholipid content as well as the carbohydrate component of Lp(a) are not constant resulting in even more aspects of Lp(a) polymorphism constituting a serious challenge for the immunochemical measurement of Lp(a) in plasma [1, 8, 9]. Results for Lp(a) levels vary from method to method depending on how the value to the calibrators were assigned and the magnitude of the impact of apo(a) size variability to the particular assay. These difficulties with Lp(a) laboratory measurement do not question the clinically relevant pathophysiological role of Lp(a) but complicate comparison of scientific study results, reliable diagnosis of Lp(a) levels, and individual classification of cardiovascular risk.

The Lp(a) threshold of 60 mg/dl had been finally fixed by the German federal authority. It must be regarded as arbitrary to a certain extent being not fully inferable by scientific data. However, it is in use to approve the clinical decision for commencing LA. Guidance is provided by the 30 mg/dl threshold often quoted with increased cardiovascular risk and by the European Consensus Panel mentioning a desirable level below the 80th percentile in Western populations, i.e., < 50 mg/dl [1, 10–12]. Alternatively for Caucasians, based on the Framingham data, a Lp(a) value of 75 nmol/l is quoted with increased risk for CVD [9]. However, an analysis from the white population in New Zealand set a similar risk threshold at 45 nmol/l [13]. Risk in male patients appears to be higher in almost all studies [12, 14].

Measurement of total Lp(a) mass in mg/dl could only be converted into a molar unit, if an accurate molecular weight would be available. The problem is that there are too many variables that play a role in converting Lp(a) values in mg/dl obtained by methods calibrated in terms of total Lp(a) mass to Lp(a) values in nmol/l obtained by methods calibrated by the World Health Organization/International Federation of Clinical Chemistry reference material [9]. There is no routine assay for determining kringle 4 type 2 repeat number in patients. Size of apo(a) is genetically determined and inversely but not exactly correlated with plasma levels. Even for apo(a) isoforms, published data on molecular weight differ substantially [15–17]. The same apo(a) allele can have different plasma levels [18]. LPA-gene exhibits heterozygosity in > 95 % of patients[19]. Expression of both alleles can be detected in 40–75 % of patients with one allele being predominantly expressed in most cases [20, 21]. Therefore, a general conversion factor from a mass unit into a molar unit cannot exist for Lp(a). The factor recommended by the *American Medical Association Manual of Style*, which was used in the Pro(a)LiFe publication, should be disregarded as erroneous [22]. According to the German reimbursement guideline, there is the need to classify patients with a Lp(a) mass threshold (i.e., > 60 mg/dl). Ranges and percentiles of Lp(a) levels for defined populations using Lp(a) assays standardized for molar number or mass of Lp(a) particles and tested in parallel series would be necessary to define population specific estimates of risk thresholds. The 80th percentile of Lp(a) levels could be a good candidate for high-risk patients (Marcovina, personal communication, [1]).

### Two-year results of the Pro(a)LiFe study

**Fig. 1:**
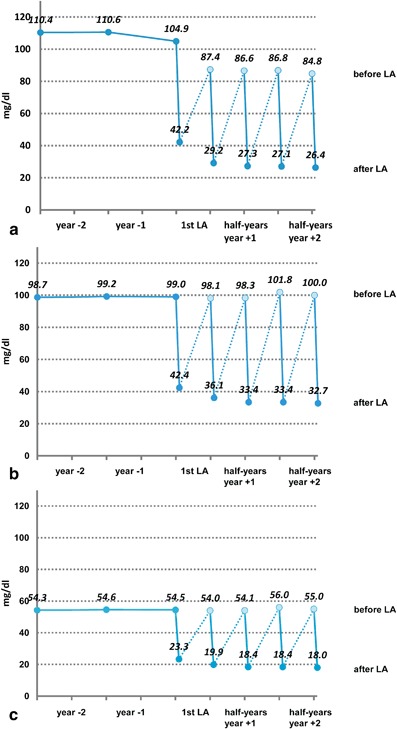
Mean plasma concentrations of lipoprotein(a) (Lp(a)) and LDL-cholesterol (LDL-C) before and after commencing chronic lipoprotein apheresis (LA) (modified according to [7]). Sawtooth-like pattern of Lp(a) and LDL-C levels illustrates weekly changes during chronic LA with approximately > 60–70 % reduction after a single treatment: **a** Lp(a), **b** LDL-C, **c** LDL-C after correction for Lp(a) cholesterol estimated as 45 % of measured LDL-C according to [8, 19]

**Fig. 2:**
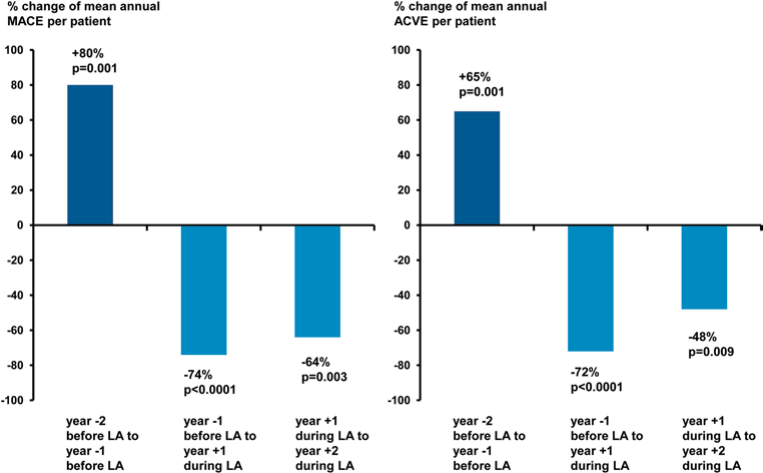
Annual percentage change of mean annual cardiovascular events during the Pro(a)LiFe study. *MACE* major adverse cardiac event, i.e., cardiovascular death, nonfatal myocardial infarction, coronary bypass surgery, percutaneous coronary intervention or stent. *ACVE* adverse cardiac or vascular events, i.e., the sum of all documented cardiac or vascular events in arterial and venous vascular beds including MACE, cerebrovascular event, peripheral vascular event, venous thrombotic event (deep venous thrombosis or pulmonary embolism) (modified from [7])

A total of 170 patients commencing LA due to Lp(a)-HLP with Lp(a) > 60 mg/dl and progressive CVD were enrolled in the Pro(a)LiFe-study. LA effectively lowered Lp(a) plasma levels by 74 % and significantly lowered incidence rates of cardiovascular events by 78 % in coronary arteries and by 74 % for all vascular beds in the 2-year blocks before and after commencing LA [7, Figs. 1 and 2]. Correcting LDL-C levels for Lp(a)-cholesterol content underlines the putative causal role of Lp(a) for the progressive course of CVD in investigated patients (Fig. 1) Analysis of mean annual event rates showed a significant increase for major adverse cardiac events (MACE) and for adverse cardiac or vascular events (ACVE) between year -2 and year -1, reflecting accelerated progression of CVD [7]. Year + 1 after commencing chronic LA was characterized by a striking reduction of MACE and ACVE rates in comparison with year − 1. Also, individual components of MACE, i.e., myocardial infarction, percutaneous coronary intervention, and coronary artery bypass graft and ACVE, e.g., cerebrovascular events followed this pattern. Significant decline of mean event rates of MACE and ACVE continued from year + 1 to year + 2 (Fig. 2). According to the identical observation periods for all patients, the mean rates directly correspond to differences in absolute numbers of events. In total, 142 MACE before LA versus 31 MACE during LA could be translated into a number needed to treat of 3 to prevent 1 MACE per patient per year for the first 2 prospective years.

### Effects of LA on atherosclerosis development and plaque morphology

The immediate effect of regular LA is characterized by pulsed physical extracorporeal elimination of plasmatic Lp(a) with its load of oxidized phospholipids and subsequent endogenous replacement by nascent Lp(a). The resulting clinical benefit of LA is preventing cardiovascular events. Atherogenesis represents a chronic process. Luminal stenosis occurs relatively late, when plaque growth outstrips the ability of the artery to compensate by expanding outward [23]. However, thrombotic complications occur suddenly and often without warning. Superficial erosion of a coronary artery with rupture of the plaque’s fibrous cap is thought to cause the majority of these severe acute complications [23]. A fibrous cap typically overlies a lipid-rich center also known as the necrotic core. Pathogenic mechanisms promoting atherosclerosis linked to elevated Lp(a) include Lp(a)-derived cholesterol entrapment in the intima, via inflammatory cell recruitment and/or via the binding of pro-inflammatory-oxidized phospholipids. Elevated Lp(a) is prothrombotic via the inhibition of fibrinolysis with enhancement of clot stabilization as well as via enhanced coagulation via the inhibition of tissue factor pathway inhibitor [1]. Ruptured plaques tend to have large lipid cores and abundant inflammatory cells. At the tissue level, improving plaque morphology could be one underlying mechanism of action for preventing clinical events by LA: quantitatively by reducing the number of vulnerable plaques and qualitatively by limiting the propensity of plaques to rupture and their thrombogenicity rather than quantitative improvement in lumen caliber [23, 24]. These changes in plaque morphology at tissue levels can be considered to confer clinical stabilization regarding the triggering of acute cardiovascular events [23].

## Conclusion

The Pro(a)LiFe-study confirmed with a prospective multicenter design that LA can be regarded as an important therapeutic approach to effectively reduce Lp(a) plasma levels and prevent cardiovascular events in high-risk patients with Lp(a)-HLP. Stabilization of atherosclerotic vascular lesions by pulsed lipoprotein elimination could be an underlying mechanism of action at tissue levels.

### Conflict of interest

For the list of authors, the following conflict of interest statements are notified: Prof. Dr. Reinhard Klingel, Dr. Cordula Fassbender, and Dr. Andreas Heibges are affiliates of the Apheresis Research Institute, which received financial support for clinical research activities by grants from Asahi Kasei Medical, Japan and Diamed, Germany.
